# Uncertainty Calculation as a Service: Integrating Cloud-Based Microservices for Enhanced Calibration and DCC Generation †

**DOI:** 10.3390/s24175651

**Published:** 2024-08-30

**Authors:** Anil Cetinkaya, M. Cagri Kaya, Erkan Danaci, Halit Oguztuzun

**Affiliations:** 1 Department of Computer Engineering, Middle East Technical University, 06800 Ankara, Türkiye; oguztuzn@ceng.metu.edu.tr; 2 Department of Computer Engineering, Iskenderun Technical University, 31200 Hatay, Türkiye; 3 Department of Computer Science and Engineering, Chalmers University of Technology, 41756 Gothenburg, Sweden; 4 RF and Microwave Laboratory, TUBITAK National Metrology Institute, 41470 Kocaeli, Türkiye; erkan.danaci@tubitak.gov.tr

**Keywords:** digital metrology, industrial internet of things, internet of measurement things, microservices, software architecture, uncertainty calculation

## Abstract

The calibration industry is renowned for its diverse and sophisticated equipment and complex processes, which necessitate innovative solutions to keep pace with rapidly advancing technology. This paper introduces an enhancement to an existing microservice-based cloud architecture, aimed at effectively managing the inherent complexity within this field. The enhanced architecture seamlessly integrates various equipment types and communication technologies, aligning diverse stakeholder expectations into a unified system that ensures efficient and accurate calibration processes. It highlights the integration of microservices to facilitate various methods of uncertainty calculation and the generation of digital calibration certificates (DCCs). A case study on RF power measurement illustrates the practical application and benefits of the enhanced architecture. Although initially focused on RF power measurement, the flexible architecture allows for future expansions to accommodate new standards and measurement techniques. The enhanced system offers a comprehensive approach to managing data flow from calibration equipment to the final generation of DCCs, utilizing cloud-based services for efficient data processing. As a future direction, this extension sets the groundwork for broader applicability across multiple measurement types, ensuring readiness for upcoming advancements in metrology.

## 1. Introduction

The continuous development of technology reshapes every sector. Demands for reducing costs, accelerating business processes, and efficiently using human resources are increasing. The metrology and calibration industry is no exception to this as it undergoes a digital transformation [1,2] to keep up with the demands of the new age. This transformation is characterized by the development and implementation of data standards and the adoption of automated and network-based solutions [3]. In this way, efficiency and data accuracy can be improved, allowing stakeholders to remain competitive in a rapidly evolving global marketplace.

Any measurement equipment has a margin of error in its measurements and may deviate over time due to various reasons, including misuse and environmental factors such as temperature and humidity. To keep this drift in the results of the equipment within acceptable limits, devices need to be calibrated regularly. For some equipment, calibration is even a legal responsibility. The device under test is checked against certain test points, and measurement results are recorded during the calibration process. A “standard device”, a trusted device usually calibrated by an upper-level body on the calibration pyramid [4], is also part of the calibration process [5] to be compared with the device under test. At the end of the process, the device under test becomes “calibrated”, and a calibration certificate is generated containing device information (such as the manufacturer and model), the calibration lab’s details, and the uncertainty values calculated by the laboratory.

The calibration process roughly involves the phases of data collection, uncertainty calculation, and calibration certificate generation. The overall process still requires the manual handling of data. Although there are tools for providing automation, they mostly focus on certain parts of the process (such as device communication and uncertainty calculation [6]), lacking an end-to-end perspective. Traditional calibration certificates are paper-based or digitally signed PDF documents, which is considered the cheapest and safest way (e.g., protected from physical errors or numerical alterations). Recently, machine-readable digital calibration certificate (DCC) standards [7] have been proposed that can ensure stakeholder satisfaction and support the ongoing digital transformation in the field. Hence, there is a need for an application to handle the calibration process holistically: starting from data collection from the calibration equipment, performing uncertainty calculations, and providing the calibration certificate. Our application addresses these expectations while adopting state-of-the-art cloud-based application development to better contribute to the digital transformation of the metrology and calibration industry.

The adoption of Internet of Things (IoT) technologies in various industries has presented new prospects in big data analytics, machine learning, and cloud computing. This concept, known as the Industrial Internet of Things (IIoT) or Industry 4.0, has delivered notable advantages such as increased productivity, shortened development cycles, swift product customization, and improved resource efficiency [8]. Recognizing these benefits, numerous IIoT solutions have been put forth in several sectors, including agriculture, manufacturing, and telecommunications. Given these advantages, IIoT technologies offer promising solutions for tackling ongoing research challenges in metrology and the calibration industry.

The notion of the “Internet of Measurement Things (IoMT)” was introduced in [9] as a layered IIoT architecture designed to segregate physical equipment, cloud-based services, and applications. This architecture builds upon the Metrology Information Infrastructure (MII) [1] initiative and draws from the experiences of the Metrology.NET platform [10]. Both MII and Metrology.NET aim to establish community-driven standards and enhance automation within the metrology field. IoMT aims to advance these efforts into an IIoT-based framework.

The IoMT architecture consists of three layers: the *physical* layer, which includes calibration equipment typically found in calibration laboratories (CLs); the *MII Cloud Services* layer, which hosts services for the calibration industry; and the *application* layer, which comprises various software used in metrology and calibration, such as calibration automation systems, asset tracking systems, and scope of accreditation (ScoA) editors.

This paper demonstrates how microservice-based solutions improve the digitalization of the metrology and calibration industry through cloud-based, scalable, and maintainable applications, ensuring data accuracy, integrity, and standardization. We developed a cloud-based application capable of handling different stakeholders’ needs and data, performing uncertainty calculations, and producing calibration certificates on the cloud. Two specialized microservices were developed to perform uncertainty calculations, each based on a different technique and implemented using different technologies and programming environments. Additionally, we integrated microservices for the generation and management of DCCs, designed with the flexibility to accommodate future extensions. We leveraged Kubernetes [11] for orchestration.

Google Kubernetes Engine (GKE) [12] utilization enhanced with variability handling brings several benefits, including automated rollouts and rollbacks, the simplified management of containerized services, and automatic scaling and load balancing capabilities. In cloud development, a rollout refers to the process of deploying a new version of an application or service. This usually involves gradually replacing the old version with the new one to ensure a smooth transition and minimal disruption. Conversely, a rollback refers to the process of reverting to a previous version of an application or service, typically to undo changes from a recent deployment that may have introduced issues or errors, ensuring the stability and continuity of the service. Therefore, GKE enables the efficient management of application updates and the maintenance of system stability, which is crucial for meeting the dynamic requirements of the calibration industry.

The previous version of the AutoRFPower [13] application was a desktop application capable of communicating with the calibration equipment, collecting measurement data from them, and calculating uncertainties based on two techniques, namely, Law of Propagation (LoP) and Monte Carlo Simulation (MCS) based on the Guide to the Expression of Uncertainty in Measurement (GUM) [14]. Danaci et al. [15] presented a comprehensive approach for uncertainty evaluation in RF power measurements using the AutoRFPower software. The software incorporates LoP and MCS methods. The study validated the software’s capability to accurately calculate measurement uncertainties and highlighted the advantages of using MCS for handling complex, non-linear relationships in uncertainty propagation.

In another previous work, a layered architecture was proposed to improve standardization and availability for the metrology and calibration industry applications in the context of the Industrial Internet of Things (IIoT), namely, the “Internet of Measurement Things” (IoMT) architecture [9,16]. Adhering to this layered architecture, the idea and initial steps of migrating the uncertainty modules of the AutoRFPower application to a cloud environment, with the name of “uncertainty-calculation-as-a-service”, were explained in another previous work [17]. In the present paper, we enhance the IoMT architecture with microservices. The AutoRFPower application is migrated to the cloud environment based on the extended architecture in the scope of this work.

Our previous work on this research direction and the contribution of this paper can be summarized as follows:

- In the previous work:
  – The IoMT architecture was presented as a specialization of IIoT architecture [9,16].
  – The AutoRFPower was developed as a desktop application to calculate uncertainties of RF power measurement devices automatically [13]. The validation of the used MCS technique was demonstrated in [15]
  – Adhering to the IoMT architecture, the idea and initial steps of migrating the uncertainty modules of the AutoRFPower application to a cloud environment were explained [17].
- In this paper:
  – The previously presented IoMT architecture is adapted to the microservice architecture.
  – Modules of the AutoRFPower application are re-implemented as microservices, including the validated MCS technique, keeping the business logic the same for the migrated ones.
  – New functionalities are added to the up-to-date version of the application as newly implemented microservices, including variability handling, DCC generation, and authentication.

In this article, an enhanced version of the work presented in [18] is demonstrated in a detailed manner. A broader background and literature review, implementation details of the uncertainty calculations, the workflows of the calibration and the authentication processes, a case study demonstrating the applicability of the approach, and the validation of the used uncertainty calculation techniques are presented for the first time in this study. Moreover, ref. [18] focused on the software architecture aspect of the proposed system; however, in the present study, we are additionally focusing on the details of the calibration processes starting from gathering data from devices such as power sensors.

The rest of the paper is organized as follows: Section 2 introduces background concepts and related work. Section 3 presents the used methodology to extend the IoMT architecture and details the overall workflow of the system along with the implementation of the components using microservices. Section 4 covers the conducted case study and discusses the proposed architecture and implementation. It also includes the results, implications, limitations, and future perspectives. Finally, Section 5 concludes the study with final remarks along with the possible future work.

## 2. Background

Metrology is defined as “the science of measurement and its applications” [19]. Calibration is an essential aspect of metrology, playing a critical role in ensuring the accuracy and quality of measurements across different domains. In time, measurement equipment can develop errors due to external factors such as misuse, temperature variations, and humidity. These discrepancies necessitate regular calibration to uphold dependable performance.

Each measurement device inherently possesses a margin of error, which is known as measurement uncertainty. The purpose of calibration is to determine this uncertainty or verify that it is within acceptable limits. This process involves comparing the measurement device against a standard to identify any deviations. While calibration identifies and quantifies these variations, the subsequent step of adjustment corrects any discrepancies. Unlike calibration, which involves testing the device, adjustment focuses on fine-tuning the device to restore its accuracy.

Measurement uncertainty is a crucial aspect of metrology and calibration, as it represents the doubt associated with the result of a measurement. It reflects the range within which the true value is expected to lie. Understanding and managing measurement uncertainty is essential for ensuring the reliability and comparability of measurement results. Factors such as the instrument’s precision, calibration method, environmental conditions, and the operator’s proficiency contribute to measurement uncertainty [14]. Accurately calculating and reporting uncertainty is vital, as it helps stakeholders make informed decisions based on measurement data, thereby reinforcing trust in the measurement process.

Quantifying uncertainty in the calibration process involves evaluating all potential sources of error and their combined effect on the measurement result. Standards such as GUM offer comprehensive frameworks for uncertainty calculation [14]. Adhering to these standards allows calibration services to ensure that their uncertainty estimates are consistent and transparent, facilitating the comparison of measurements across different laboratories and applications.

While LoP is commonly used for calculating uncertainty, the MCS method offers a versatile and robust approach for uncertainty calculation in calibration processes. Unlike the strictly defined analytical approach of LoP, MCS uses computational algorithms to model and propagate uncertainties through a large number of simulated trials. This stochastic method excels in handling complex and non-linear relationships that may be difficult to address analytically, providing a detailed statistical representation of measurement uncertainties by generating and analyzing numerous random samples [20].

In the realm of software architecture, a prominent approach for organizing software applications into a suite of services is service-oriented architecture (SoA), and microservices are a modular way of realizing SoA. SoA prioritizes the creation of loosely coupled, interoperable services that communicate over a network using standard protocols. This approach allows for modular design, enabling the reuse and integration of services into larger systems with ease. SoA examples include web services, which facilitate diverse applications to exchange data, and enterprise services, which streamline business operations across different platforms [21].

Microservices represent an evolution of the SoA paradigm, focusing on constructing software applications from a collection of small, self-contained, and independently manageable services. The aim is to divide applications into smaller, independently deployable services, each assigned to a specific business task. These services interact through lightweight, standardized protocols, ensuring seamless communication despite technological diversity. This modular approach allows for the utilization of various programming languages, databases, and technology stacks, as long as they adhere to common interfaces and protocols. This architectural strategy enhances scalability, agility, and maintainability, empowering teams to deploy updates and new features more frequently and reliably [22]. Additionally, microservices are typically designed to be stateless, which means that each client request is treated as an independent transaction, without relying on prior interactions. This stateless approach enhances scalability and resilience, allowing services to handle requests independently and be easily replicated across different environments. Both SoA and microservices contribute to the overall objective of creating flexible, maintainable, and scalable software systems by leveraging modular service composition.

Variability in systems and software engineering refers to the ability of a system or software product line to be efficiently extended, changed, customized, or configured for use in a particular context, enabling the creation of different product variants that share a common core but differ in certain aspects to meet specific customer requirements or market demands [23].

Variability management plays a crucial role in software engineering by facilitating the efficient development and maintenance of a range of related software products. Effective variability management involves the identification, modeling, and management of the commonalities and differences among products. This process is essential for optimizing reuse and ensuring flexibility in product customization. Pohl et al. [24] emphasized the significance of variability mechanisms, such as feature models, decision models, and configuration management, in systematically addressing the complexities associated with product variations. By leveraging these mechanisms, organizations can strike a balance between standardization and customization, thereby reducing time to market and enhancing product quality.

### Related Work

To the best of our knowledge, our approach is one of the pioneering efforts to address the calibration process holistically. Existing studies tend to focus on specific aspects or phases of calibration. As a result, there are only a limited number of studies available for comparison.

**Figure 1:**
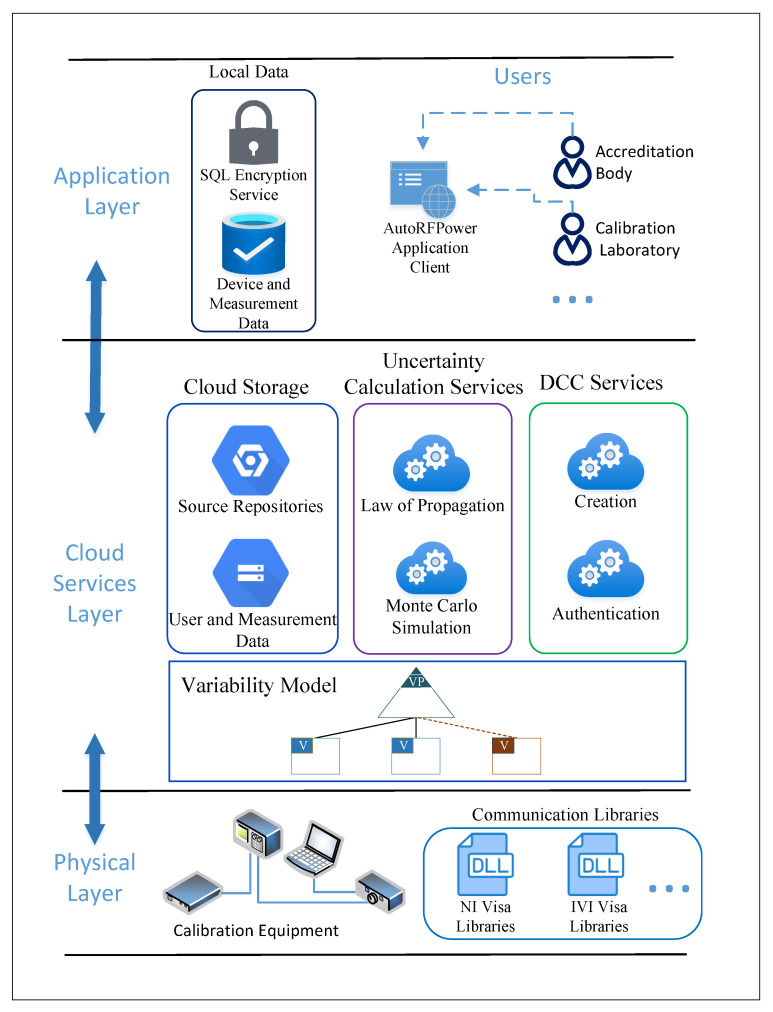
The IoMT-compliant architecture of the system and its components.

IoMT was presented as a layered IIoT architecture for metrology and calibration industry applications [16]. The three layers are composed of the physical layer, the cloud services layer, and the application layer as shown in Figure 1. When the architecture was initially proposed, the cloud services layer lacked a microservices vision and corresponding implementations. This work enhances the IoMT architecture with the microservices perspective, and its applicability is shown with the AutoRFPower application.

Zet et al. [25] described an automated process for calibration and DCC generation using blockchain technology to improve accessibility among stakeholders. In contrast, our system utilizes cloud technologies, offering enhanced flexibility and scalability. Our incorporates microservices to integrate various measurement types, reducing interoperability issues. This architecture supports the easy integration of different standards and measurements, without being constrained by the inherent limitations of blockchain technology.

Oppermann et al. [26] introduced the “operation layer” at PTB (Physikalisch-Technische Bundesanstalt) to enhance domain workflows using a cloud-native, distributed microservices architecture. This layer streamlines processes, breaks down data silos, and automates the creation of DCCs by connecting laboratory workflows with administrative data. While their approach provides a broad solution for managing and improving workflows in the domain, including the DCC workflows, we propose a more focused tool for the calibration process, providing details at the implementation level.

Pontarolli et al. [27] conducted a study in the field of Industrial Automation Systems titled “Microservice-Oriented Architecture for Industry 4.0”. Their focus was on using microservices to enhance industrial applications. They implemented their approach using the Moleculer framework [28], an open-source microservices framework for Node.js. The study demonstrates the significant benefits of microservice architectures in integrating advanced technologies such as IoT and cloud computing within industrial settings. While Pontarolli’s work addresses general industrial automation, our approach specifically targets the calibration industry. We integrate various equipment types and communication technologies to ensure efficient and accurate calibration processes. Additionally, our architecture utilizes a Continuous Integration/Continuous Delivery (CI/CD) pipeline enhanced with a Textual Variability Model (TVM) for dynamic product configuration to ensure the ability to adapt to changes while boosting the system’s responsiveness.

Microservices Developer (MSDeveloper) [29] aims to establish a domain-oriented development environment by integrating feature and process models into a well-defined architectural framework. This approach utilizes variability management for product configuration, employing a feature model-driven methodology and microservices. While MSDeveloper is crafted as a domain-oriented development environment by integrating feature and process models into a layered architecture, our approach is specifically designed to tackle the unique challenges in the calibration industry. By incorporating a TVM, our architecture dynamically manages the heterogeneity of calibration equipment and processes. This model enables real-time adjustments to calibration settings and hardware configurations. Integrated into a microservice-based cloud architecture, this approach not only enhances system flexibility and scalability but also efficiently manages data flows and the generation of digital calibration certificates (DCCs), providing a streamlined and adaptable solution for the metrology domain.

The study conducted by Nummiluikki et al. [30] discussed the implementation of DCCs within an industrial setting. Their research is a part of a proof-of-concept project involving multiple partners, aimed to test the feasibility of DCCs in a fully digitalized calibration environment. The project focused on creating a digital environment for calibration data generation, transfer, and usage, including DCC validation and digital signatures. Although their approach effectively demonstrated a digitalized calibration process, our proposed architecture extends this concept by integrating uncertainty calculation services to work alongside the implemented DCC generation/authentication services via leveraging microservices to enhance scalability and flexibility.

The cloud-side implementation of AutoRFPower contains two main services: uncertainty calculation and DCC generation. There is related work making uncertainty calculations available online, such as the NIST Uncertainty Machine (NUM) [6]. NUM is an online tool based on a client–server architecture that performs uncertainty calculations based on LoP and MCS. Unlike our approach, which integrates measurements from physical equipment and generates DCCs in an IIoT environment, NUM focuses on server-side calculations without considering hardware-level measurements or producing DCCs. Another example is Metas.UncLib [31]. It is a standalone desktop application that can be used to solve complex problems related to metrology, so it is not comparable with our approach.

A DCC is the digital version of a calibration certificate. Since the calibration certificate is an important output of the process [17], it circulates among the stakeholders in the industry, including customers, calibration labs, and national metrology institutes. Therefore, digitizing and standardizing this document is crucial. This effort is pioneered by PTB by providing a structured format for DCCs [4,7]. We aim to generate PTB DCC-compatible calibration certificates and currently adopting their standard as much as possible. On the other hand, our architecture is designed with extendibility in mind, so we can incorporate new standards if and when they emerge.

## 3. Methodology

The IoMT architecture is extended by adopting a microservice perspective and related technologies on the cloud layer. Figure 1 represents the IoMT-compliant architecture of the system detailing the entities residing in the layers.

The application layer hosts the user interface (UI) along with essential components that facilitate communication with the hardware in the lower layer. These components include device-specific drivers. Additionally, certificate information for the registered devices is collected from users and securely stored in an encrypted database. Communication with measurement devices is facilitated through these device drivers in the application layer, which interact with communication libraries located in the lower layers. The results of the measurements are then stored in the SQL database within the application layer.

The cloud services layer contains the uncertainty calculation services, the DCC services, and the cloud storage services. Both measurement and user data are hosted on Microsoft SQL Server in a container, ensuring high availability and secure data handling practices. Specifically, our architecture utilizes advanced encryption and access control mechanisms to secure data transmission and storage, while also facilitating uncertainty calculations and digital certificate generation. This layer not only supports high data throughput through the use of purpose-built microservice containers but also interacts seamlessly with lower layers, enabling the product configuration using variability models to be resolved and integrated across the system.

The physical layer contains the measurement setups, featuring a PC configured to control and process data generated by the registered devices, such as a power signal generator, a power meter, a power sensor, and an optional attenuator. All components in this layer work in coordination to enable precise measurement and calibration.

**Figure 2:**
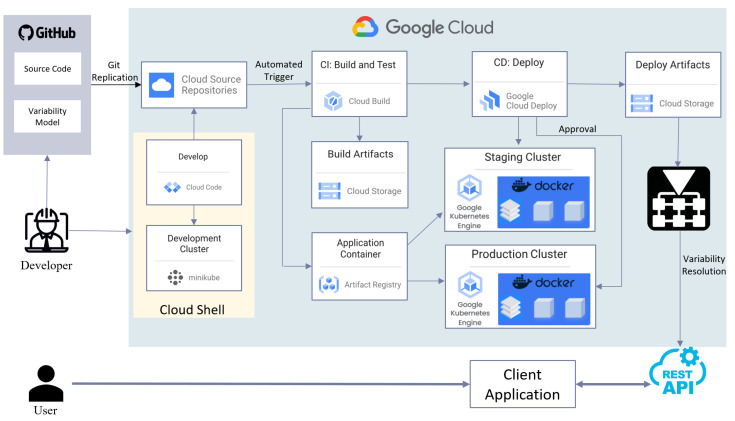
The system architecture on the Google Cloud platform and the CI/CD pipeline (adapted from [32]).

Figure 2 demonstrates the Google Cloud architecture of the proposed system. We adopt and adapt the Google Cloud platform’s recommended workflow for the development and deployment of containerized applications, utilizing a CI/CD pipeline [32]. This architecture enables a full-cycle development workflow that begins with collecting measurements from the hardware at the lowest level, transferring this data to the cloud environment, performing uncertainty calculations, and ultimately generating DCCs in compliance with standards set by regulatory bodies such as PTB.

There are three core Google Cloud services at the core of our CI/CD pipeline: Cloud Build, Artifact Registry, and Cloud Deploy. Cloud Build is a service that executes builds on the Google Cloud infrastructure. It compiles source code, performs tests, and produces ready-to-deploy software packages. Artifact Registry is designed to store, manage, and secure the container images and additional libraries. It facilitates consistent access control and integration with existing CI/CD tools by providing a centralized location for the custom-built software artifacts. Finally, Cloud Deploy automates the delivery of applications to specified Google Cloud environments. This service streamlines deployments, ensuring they are repeatable, predictable, and secure across multiple stages of the production process. The continuous aspect of CI/CD is crucial for our application, allowing for seamless and frequent updates without disrupting the system. This capability is vital since the addition of new devices and features is frequent, and any disruption during the updates may result in downtime or loss of data integrity, which is unacceptable in calibration processes. Combined, these services automate the software deployment process from initial build to final deployment, improving the efficiency and reliability of the system’s software development lifecycle.

In addition to the conventional workflow, an automated trigger has been integrated into the Cloud Build service. YAML [33], a human-readable data serialization language, is used to handle the manipulation of configuration files. This trigger is executed whenever a Git replication occurs from a source Git repository to the Cloud Source Repository. In this way, every time when a change is made in the Google Source Repository or an update is committed via Git replication, a new build for the updated version is directly generated and staged under the deployment cluster ready to be deployed after approval without any interruption to the running instance.

Users of AutoRFPower are calibration laboratories that use different families of equipment having diverse communication protocols and libraries. This diversity is addressed by variations in code, hence the need for modeling and managing variability. Therefore, a customization mechanism is needed. Rather than performing the customization in an ad hoc manner, we employ a systematic way of handling variability. To this end, an additional TVM is developed and integrated into the source code. This TVM manages essential information such as device manufacturer/models for all device types, compatibility information among those devices and communication protocols/libraries, and access tokens for all microservices offered across the system. On the other hand, each client/user has a configuration file that includes the variants/features assigned to them, regulating their permissions to access system components, various devices, and functions.

The interaction between the Cloud Services layer and the application layer is facilitated through the exchange of JSON-formatted files over HTTP requests via RESTful API [34]. This approach provides a standardized and stateless (session-independent) communication protocol that simplifies the integration of various services and enhances the scalability and maintainability of the system. Additionally, the utilization of RESTful principles allows our architecture to maintain efficient data exchange and real-time updates. For example, transfer of the mentioned configuration information from the client and the resulting response from the cloud services layer which is generated according to the TVM allow the existing varibility to be resolved (dynamically) in run time. Additionally, since the TVM is embedded into the source code, any changes made in this model trigger a new build which can be deployed without interruptions to the running services.

Therefore, our approach, including automated triggers and variability resolution, enhances the processing of calibration data and the creation of DCCs by increasing the system’s responsiveness and allowing the seamless integration of new services or modifications without significant disruptions.

In the current implementation of our system, we generate microservices for two different uncertainty calculation methods: LoP and MCS. Although the formulations of these calculations are provided by the International Bureau of Weights and Measures (BIPM) [14], there is no restriction on the tools and technologies that can be used for their implementation. Therefore, there are different programming languages and environments in their implementations, creating heterogeneity. Given the fact that the calibration equipment requires specific libraries for communication, each technique can run in its own environment. Hence, having microservices for these implementations and the containerization of them is a promising solution. For example, in our application, the LoP service is implemented using the C# programming language and its native libraries, while the MCS service is developed in Python 3, utilizing the open-source Pandas data analysis library. Moreover, the calibration equipment has its own environment encapsulated in the containers.

**Figure 3:**
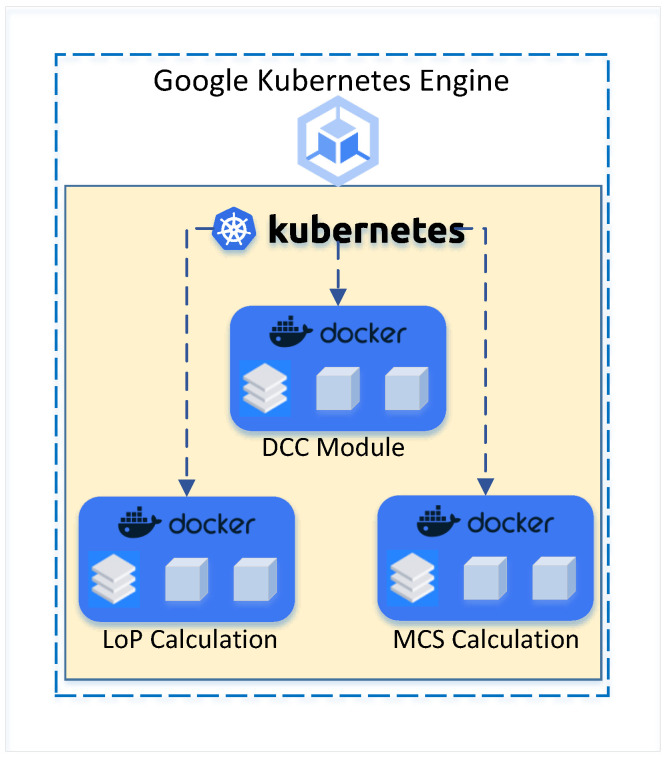
Dockerized containers in the cloud environment.

Figure 3 presents the dockerized components to host various microservices within the cloud environment, which are orchestrated via the Google Kubernetes Engine. Each microservice is encapsulated within its docker container, thus ensuring isolation while lowering dependencies, enhancing scalability, and increasing deployment speed. Furthermore, the adoption of Docker containers to host microservices has many advantages such as shortening the development time, improved fault tolerance, and providing a more consistent environment during the development, testing, and production [35]. Inclusion of the GKE for microservice orchestration ensures efficient resource management, automatic scaling and robust load balancing capabilities especially crucial for computationally heavy tasks such as MCSs. This configuration is not only useful for the optimization of operational efficiency but also enhances the reliability and adaptability of the system, accommodating agile responses to dynamic requests caused by rapidly advancing technology and industry’s demands.

In the DCC services microservice, we employ the XML data format in compliance with the PTB standardization of DCCs. Using XML facilitates well-structured and highly exchangeable data that support seamless transitions when formatting DCCs based on the existing definitions and adapting to newly designed standards. After performing the uncertainty calculations for either or both LoP and MCS microservices, the results are converted into the XML format and forwarded to the DCC microservice, with the utilization of REST API [34] and the Flask framework [36]. Then, this XML file is parsed and converted into a human-readable PDF format in the cloud environment where users can access their certificates through an authentication service.

We implement a robust method for authenticating DCCs by leveraging UUIDs (Universally Unique Identifiers). These UUIDs are generated using a combination of the current timestamp and the Media Access Control (MAC) address of the machine, ensuring both temporal and spatial uniqueness. Each original document stored on the cloud storage is assigned a unique UUID, which is then used to generate a corresponding quick response (QR) code. When scanned, this QR code directly links to the original document on the cloud server, ensuring its authenticity. By embedding these QR codes in our DCCs, we provide a reliable means for verifying the integrity and originality of the documents. This method enhances security by significantly reducing the probability of duplication, as the identifiers are both time- and hardware specific. Implementing UUID-based QR codes thus ensures a high level of trust and reliability in the DCC authentication system.

### 3.1. System Workflow

This section provides a comprehensive overview of the RF power measurement implementation, covering the entire process from initial measurements taken in the physical layer (test setup) to uncertainty calculations in the cloud services, and concluding with the generation of DCCs in a cloud-based environment. This end-to-end approach utilizes an enhanced microservice architecture in support of IoMT to guarantee efficient and accurate calibration processes.

**Figure 4:**
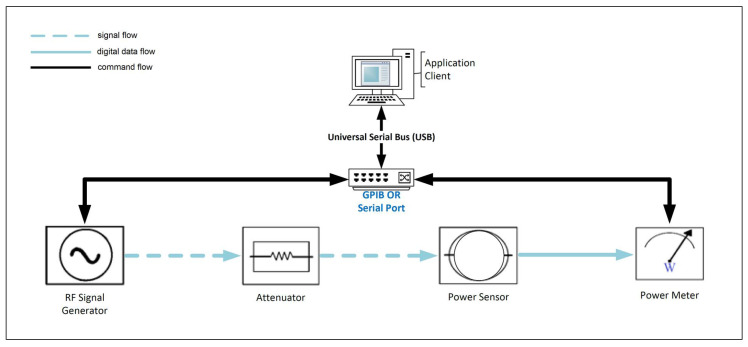
The measurement setup and its connections.

The process begins with the user (e.g., a technician or engineer) connecting the test setup, which consists of a signal generator, power sensor, power meter and an optional attenuator to a computer running the AutoRFPower (client) application. This application can communicate with the test setup using both General Purpose Interface Bus (GPIB) adapters and serial communication ports via the Institute of Electrical and Electronics Engineers (IEEE) 488.2 communication protocol. A schematic of the test setup is shown in Figure 4. The necessary device drivers (such as NI Max, Keysight IO Suite, etc.) must be installed on the PC running the software.

**Figure 5:**
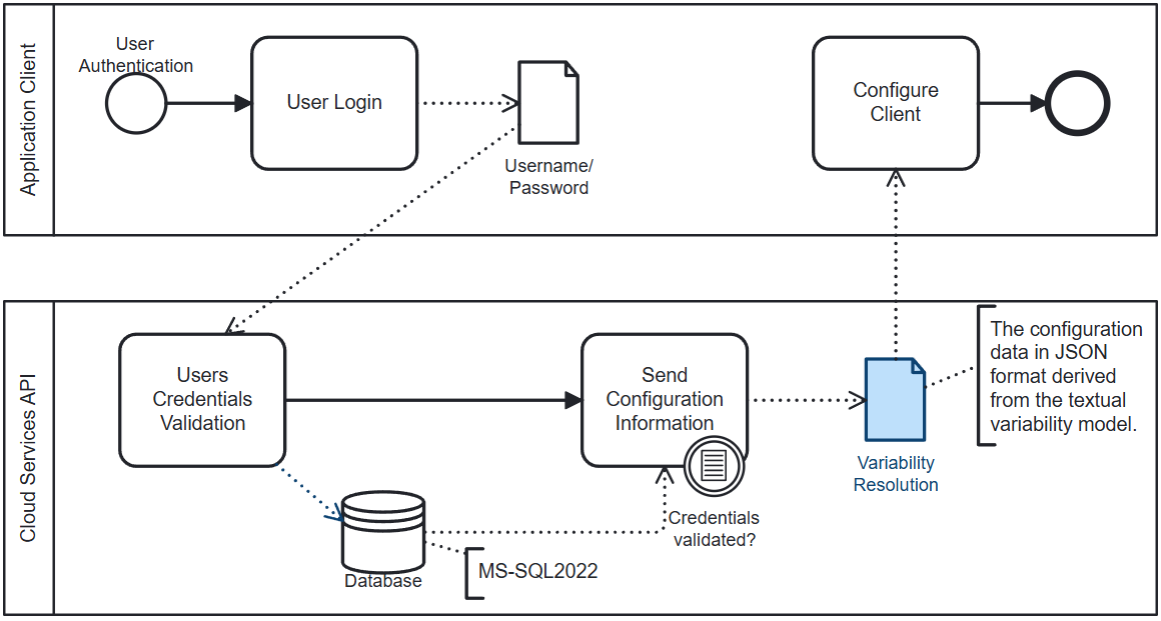
User authentication and client configuration based on variability resolution.

The process model shown in Figure 5, drawn using BPMN (Business Process Model and Notation) 2.0, illustrates the user authentication and client configuration based on variability resolution. BPMN 2.0 is a graphical representation for specifying business processes in a business process model. It provides a standard way to visualize the steps in a process, which enhances clarity and communication among stakeholders.

When the devices are prepared for measurement, the operator launches the client application from the computer, which is connected to test devices using a GPIB adapter. The application starts with a login window and prompts the operator to enter their credentials. Then, the username and passport entered are forwarded to the Cloud Services API (CSAPI) and compared with the stored credentials. If access is granted by the system, CSAPI returns a configuration file in JSON format. This file consists of information derived and transformed from a TVM, which is used for the configuration of the client application based on the features and devices that are allowed for that specific user. The access rights for users can be updated based on their qualifications by a system admin or an authorized person.

**Figure 6:**
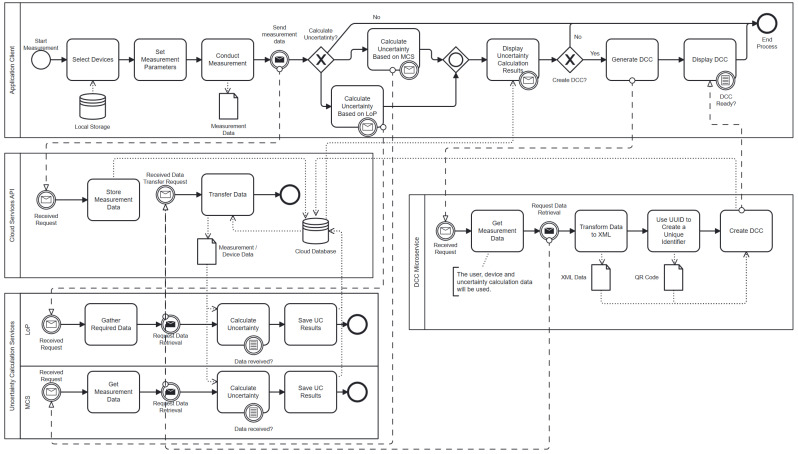
The process for the uncertainty calculation and DCC generation.

In a technology-intensive domain such as metrology, it is of the utmost importance to ensure that only qualified personnel are authorized to operate specific equipment and carry out designated tasks. Inadequate qualifications can result in significant errors and potential misconduct, posing a risk to the integrity of the calibration process. Therefore, the effective management of user access is essential. By restricting device operations and feature access based on user qualifications, the system guarantees that only trained and certified individuals can execute specific functions. This approach not only enhances the precision and reliability of the calibration process but also ensures adherence to industry standards and regulations. Upon finalizing the client configuration, the operator can initiate the measurement process. The process model illustrated in Figure 6 outlines the overall workflow of the system. It begins with automated RF power measurements on the client application and demonstrates the implemented microservices for uncertainty calculations and DCC operations, as well as the interactions among all these components. This model offers a comprehensive understanding of the system’s end-to-end operations, promoting clarity in the sequence of actions and the roles of various components.

Automated RF power measurement commences with the operator selecting from registered devices stored in the local database. If any device from the measurement setup has not been registered, the operator registers that device to the system by entering specific information about the device, such as serial number, certificate information, and any other required details for the uncertainty calculations. The process then continues with the determination of the measurement parameters. These parameters include the test points (power levels and frequencies to be measured), the number of measurements for each test point, and the waiting times between instances.

When the measurement is completed, all results, along with the setup and operator information, are combined, converted into a JSON file, and sent to the CSAPI with an HTTP request to be stored in a cloud database.

### 3.2. Statistical Techniques Used

Our system offers users the flexibility to conduct uncertainty calculations using either the LoP or MCS methods. With a simple click, users can trigger their chosen method, which then sends a request to the CSAPI to gather the essential data for the calculations. Once the data are transferred to the designated container, the calculations are executed, and the results are subsequently showcased in the application and stored in the cloud database within the cloud services container.

The LoP method, which is based on the principles outlined in the specifications provided in reference [14], utilizes analytical formulas to systematically evaluate the uncertainties associated with various measurement processes. This method provides a structured and standardized approach, making it widely utilized in various calibration practices. The implementation of this method is straightforward, as the core principles of GUM are consistent and universally applicable.

In the present study, the LoP method based on GUM is integrated into the cloud environment to handle uncertainty calculations for various calibration processes. The implementation follows the same principles, ensuring that all input parameters contributing to the uncertainty are accurately transformed into normal distributions. The combined uncertainty is calculated using the following Equation (1):

(1) $$u\left(k=1\right)=\sqrt{\sum_{i=1}^{n}\left(c_{i}^{2}\cdot u_{i}^{2}\right)}$$

where u(k=1) is the combined uncertainty with coverage factor one (68 % reliability), $c_{i}$ represents the sensitivity coefficient of each uncertainty component, and $u_{i}$ denotes the uncertainty value of each component.

MCS utilizes computational algorithms to model and propagate uncertainties through a large number of simulated trials, making it a stochastic method. By generating and analyzing numerous random samples, MCS provides a detailed statistical representation of measurement uncertainties. Its flexibility enables tailored application to specific scenarios and measurement complexities, making it a powerful tool in modern metrology. However, this flexibility also means that the MCS implementation can vary significantly from case to case, depending on the specific requirements and available computational resources. This adaptability is a key advantage of MCS, allowing for precise and customized uncertainty assessments across diverse calibration contexts.

In the present study, the MCS method was adapted to the cloud-based system, allowing for the simulation of uncertainty calculations using large datasets and extensive computational resources. The combined uncertainty was calculated using Equation (2):

(2) $$u\left(k=1\right)=\sqrt{\sum_{i=1}^{n}u_{Ri}^{2}}$$

where u(k=1) is the combined uncertainty with coverage factor one (68% reliability), and $u_{Ri}$ represents the randomly generated uncertainty values of each component.

**Figure 7:**
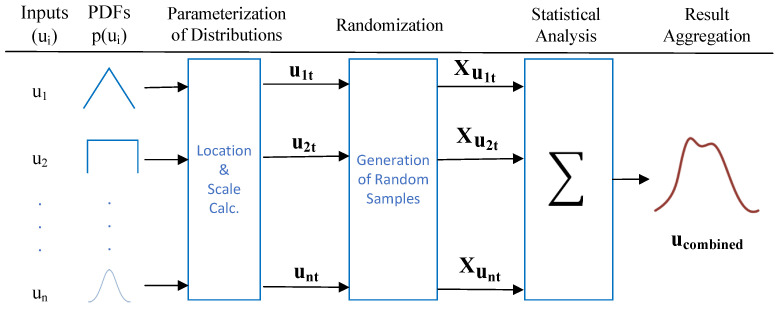
Monte Carlo Simulation process for uncertainty calculations.

The MCS process flow for RF power measurement uncertainty is illustrated in Figure 7, providing a clear and structured visualization of the steps involved in evaluating uncertainty based on MCS in RF power measurements. This figure is essential for understanding the systematic flow of the algorithm and how each component contributes to the overall uncertainty analysis. The workflow begins with inputs $\left(u_{i}\right)$, representing the initial data or parameters required for the simulation. These inputs are then transformed into Probability Density Functions (PDFs) $p(u_{i})$, defining the underlying distributions that model the uncertainties.

Next, the process moves to the parameterization of distributions stage, where the location and scale parameters are calculated. The location parameter typically indicates the central tendency of the distribution, such as the mean or expected value, while the scale parameter reflects the dispersion or spread, such as the standard deviation. These parameters are crucial, as they define the shape and characteristics of the probability distributions used in subsequent steps. In the randomization stage, $10^{5}$ random samples are generated for each distribution based on the previously defined parameters. This extensive sampling ensures a robust representation of the range of possible outcomes, allowing for a thorough analysis of uncertainty. The samples then undergo statistical analysis, where key metrics, including mean, standard deviation, and confidence intervals, are calculated to quantify the uncertainty in the system. Finally, in the result aggregation step, these statistical results are combined into a single, unified measure of uncertainty, referred to as $u_{\mathrm{combined}}$.

**Figure 8:**
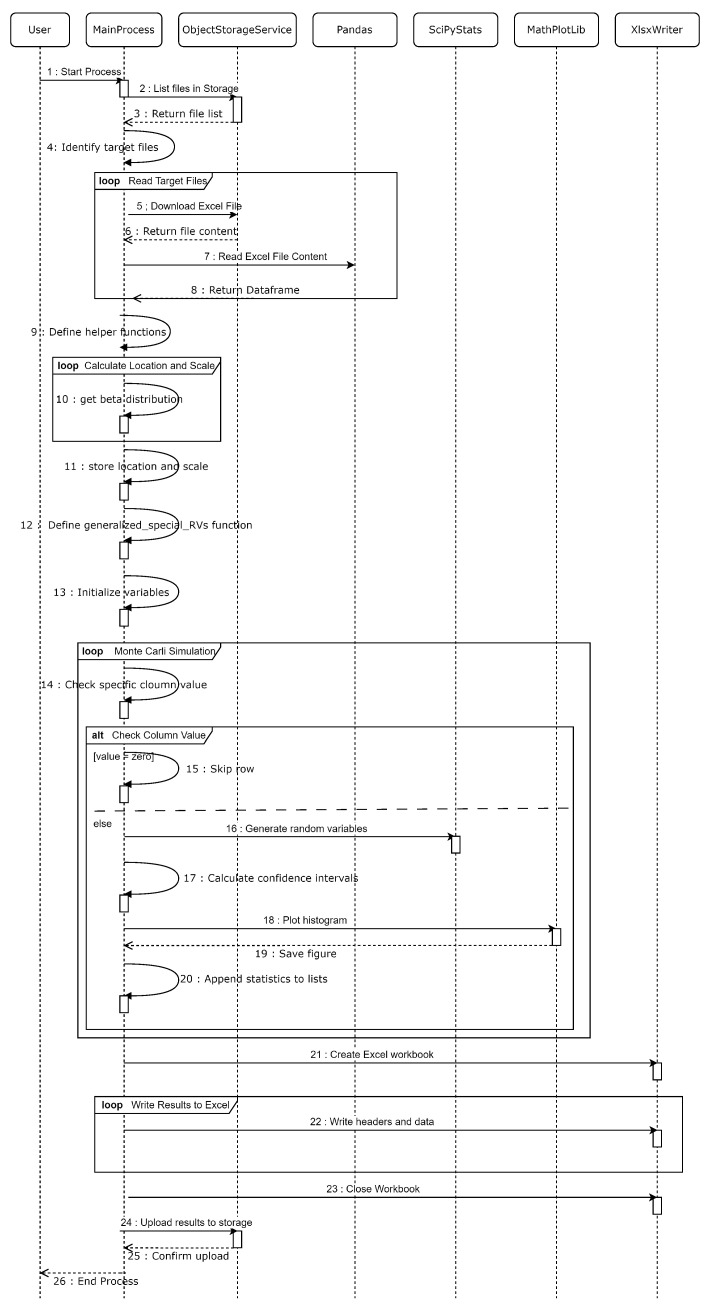
Sequence diagram for the Monte Carlo Simulation.

Expanding on the detailed process flow outlined in Figure 7, we will delve deeper into the technical implementation of the MCS algorithm, especially when deployed as a container in a cloud environment. To gain a more granular understanding of how the MCS algorithm operates within this context, we present a Unified Modeling Language (UML) [37] sequence diagram in Figure 8. This diagram complements the process flow by illustrating the specific interactions and integrations within the MCS container, showcasing the algorithmic flow as it interfaces with cloud-based services.

The sequence diagram details the steps involved in quantifying uncertainties by simulating various potential outcomes based on input data. The MCS algorithm is implemented through several key stages: initializing variables, setting up necessary parameters, reading and processing input data files stored in cloud-based object storage, generating random variables to model uncertainties in the measurements, calculating confidence intervals for the simulated data, and saving the results back to the cloud storage for further analysis and reporting.

The sequence diagram for the MCS algorithm includes several key participants and interactions. The user initiates the simulation process, triggering the main process which orchestrates the entire simulation. The main process interacts with the object storage service, a cloud-based service used to store and retrieve data files. Pandas, a data manipulation library, is employed for reading and processing Excel files, while Matplotlib, a plotting library, is utilized for visualizing the results. Scipy.stats, a statistical library, generates random variables necessary for the simulation, and XlsxWriter, a library for writing output results to Excel files, stores the simulation results. Each of these components works seamlessly together to execute the MCS algorithm, ensuring efficient data handling, statistical computation, and result visualization and storage.

The process begins with the user initiating the main process, which requests a list of files from the object storage service. This entails querying the cloud storage for files that match a specific naming pattern recognized as an output of the client application. Once the target files are identified, the main process downloads the content of each file from the object storage service and reads it into a Pandas DataFrame, which serves as the primary data structure for managing and processing the input data. Additionally, the main process defines various helper functions, including get_beta_distribution, which calculates the statistical parameters necessary for the simulation.

The algorithm calculates the location and scale parameters for the beta distribution by iterating through the input data and stores these parameters for use in subsequent steps of the simulation. The generalized_special_RVs function is defined to generate random variables based on the input data and various statistical distributions essential for modeling the uncertainties in RF power measurements.

The process continues with the MCS loop iterating through each row of the input data. If a specific column value is zero, the row is skipped to avoid unnecessary computations. For other rows, the algorithm generates random variables using the defined function, calculates confidence intervals, and plots histograms using Matplotlib. These histograms offer a visual representation of the probability distribution of the simulated data. The calculated statistics, including mean and standard deviation, are then appended to respective lists for further analysis.

Upon completion of the simulation loop, the main process creates an Excel workbook using XlsxWriter, where it writes the results, including confidence intervals, mean values, and standard deviations. The completed Excel workbook is then uploaded back to the object storage service to ensure secure storage and accessibility for further analysis. The process concludes with the main process printing the total simulation time, marking the end of the MCS algorithm’s execution. This series of interactions and computations effectively models the uncertainties in RF power measurements, providing valuable insights into the system’s behavior under various conditions.

Finally, the operators can generate DCCs based on the performed measurements and subsequent uncertainty calculations by sending a request to the DCC microservice via the application UI. When triggered, the DCC microservice container retrieves the necessary data from the CSAPI. These data include the user information based on the logged-in profile, all of the data on the devices selected in order to perform the measurement and uncertainty calculation results. The data are transformed into XML format inside the container to be shaped in compliance with the defined specifications/standards. Then, the XML data are embedded into HTML tags to form a document which can be distributed/printed in “.pdf” format.

To ensure the created document can be authenticated by officials or third parties, a method called “UUID Generator” is called within the container. This method uses a combination of the current timestamp and the MAC address of the device to create a unique 32-character string that is difficult to replicate. This unique code is appended to a prefix link that points to a running instance of the authentication service on cloud servers. A QR code containing this link is placed on each DCC, allowing each certificate to be authenticated using the cloud authentication service.

## 4. Discussion and Results

This section presents a comprehensive evaluation of our system, commencing with a detailed case study that illustrates its ability to integrate and manage measurement devices, conduct measurements, and perform uncertainty calculations within a cloud-based environment. Subsequently, we validate the accuracy and reliability of the uncertainty calculation methods by comparing them with results from a prior study [15] conducted in local settings. Additionally, the section includes a critical evaluation of the employed uncertainty calculation methods, namely, LoP and MCS, highlighting their respective strengths and limitations. Finally, we discuss the broader implications of our findings, evaluate the system’s constraints, and suggest future avenues for enhancing the system’s capabilities, particularly in the realm of digital metrology and calibration.

### 4.1. Case Study: A Practical Application of the System

**Figure 9:**
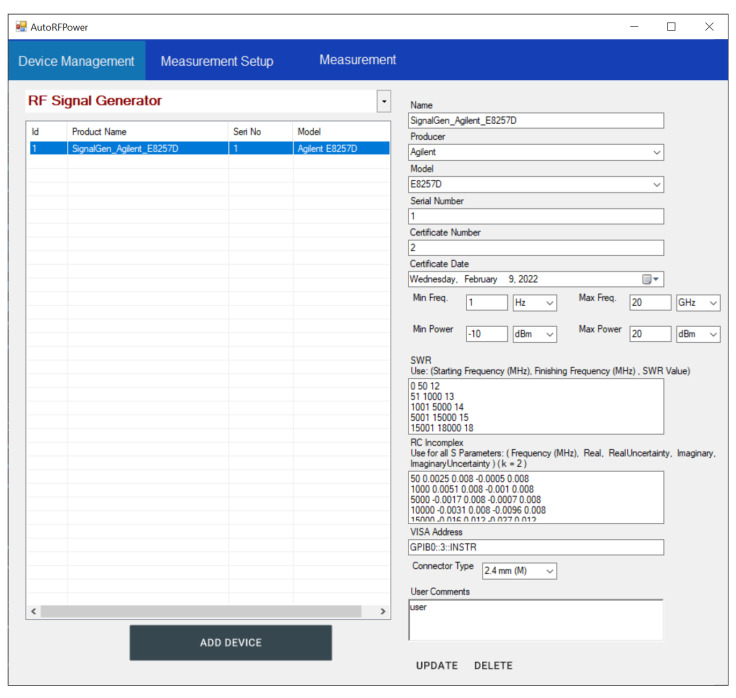
The device management user interface of the client application.

An Agilent E8257D signal generator, a Keysight 8481A power sensor, and a Keysight N1914A power meter were connected using a National Instruments (NI) GPIB adapter. The client application was started, and user credentials were entered to access the system. At this point, the client was configured in real-time according to the granted access rights based on the application of the TVM for our user. After configuration, only the devices available to us could be listed and selected. We checked the registered devices and realized that the signal generator had not been registered in the system yet. Therefore, we registered it (please note that the serial number and certificate number fields are displayed with placeholders for confidentiality reasons). The device management form is shown in Figure 9.

Next, we opened the NI Max application to verify the communication addresses assigned by the operating system for the signal generator and power meter. Since these addresses are not permanent and may change based on the virtual environment of the computer (e.g., after each restart), we compared them and updated as necessary. Then, we moved on to the measurement setup interface and chose the devices to be used for the measurements. Once selected, we were prompted to enter the measurement parameters in the measurement form. We designated 24 test points for the measurements, including test frequencies of 50, 1000, 5000, 10,000, 15,000, and 18,000 MHz, and power levels of 0, 5, 10, and 15 dBm. We also set the waiting times between measurements. Afterwards, we added remarks for the test and documented the environmental conditions: temperature in degree of Celsius and humidity as a percentage. The measurement was then initiated and executed as per the configuration. Upon completion, the results were displayed on the user interface and saved to the cloud database.

**Figure 10:**
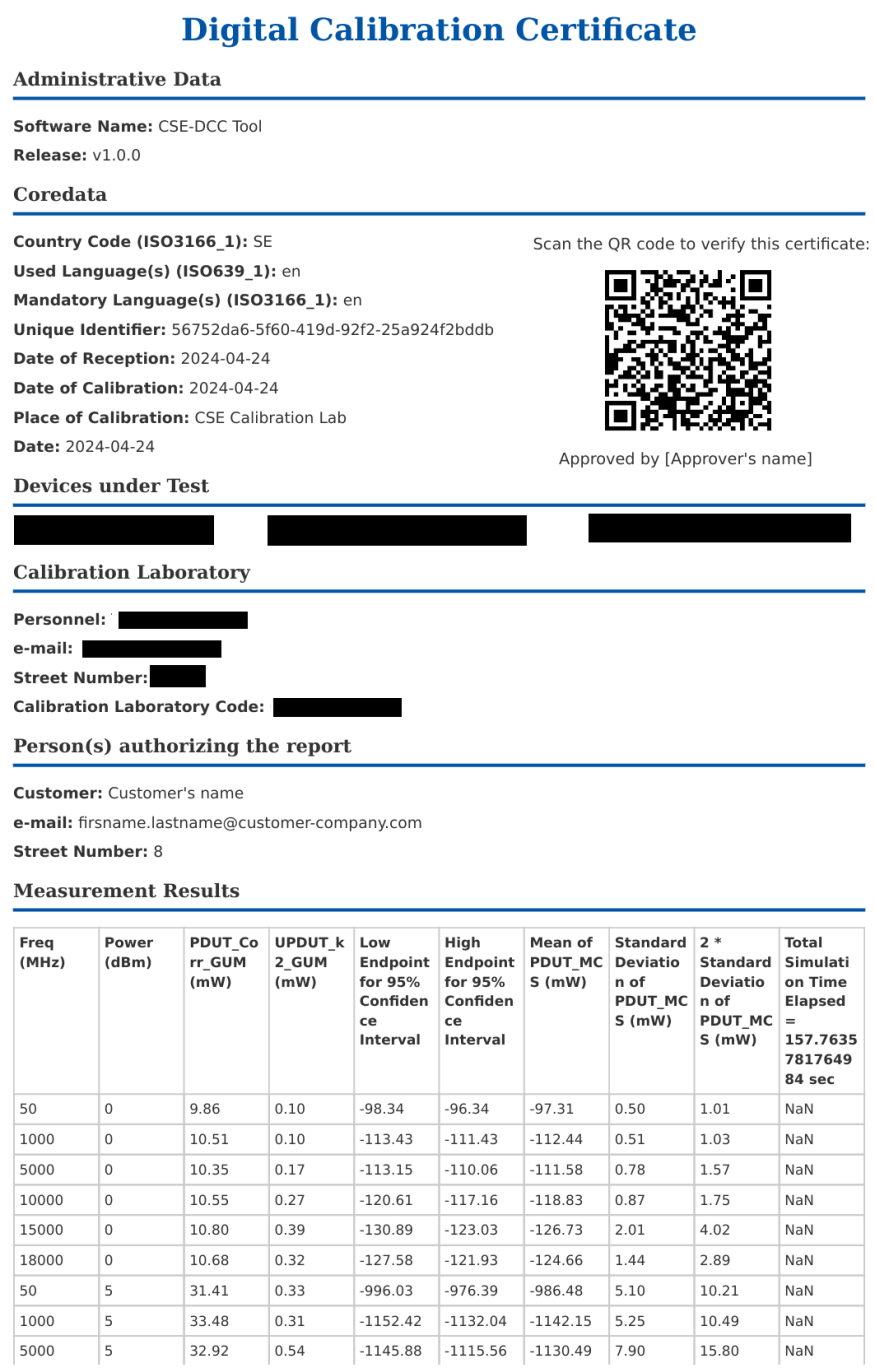
An excerpt of the generated DCC.

After completing the measurement, we initiated the uncertainty calculations using a designated button. The MCS calculations were carried out in the cloud environment and took approximately 20 min to process data from the 24 chosen test points. Upon completion, we received a link to an Excel file containing the results. Subsequently, we proceeded with the generation of the DCC. An excerpt from the generated DCC is illustrated in Figure 10. We then used a camera to scan the QR code, which directed us to a web page displaying the original results of the MCS calculations.

### 4.2. Validation of the Uncertainty Calculations

In order to ensure the reliability and accuracy of the uncertainty calculation methods integrated into our microservice-based cloud architecture, we cross-validated them with the LoP and MCS methods from our prior research [15]. These methods were originally validated through a comprehensive comparison of uncertainty calculations performed using the AutoRFPower software and the Oracle Crystal Ball simulation application in a local environment. The study tested the methods across various RF power levels and frequencies, demonstrating their effectiveness in practical applications. This section outlines the validation process for both methods after migrating them to the cloud environment as microservices. Despite the initial validation being conducted locally, the methods are expected to function equivalently in the cloud environment, as they are dockerized versions of the same codes.

The LoP method [14] calculates the combined uncertainty by assuming that all input parameters have normal distributions. In our previous study, we implemented this method in the AutoRFPower software, which was validated by comparing manually calculated uncertainties using MS Excel with those generated by the software. The results showed a high level of agreement, with differences at the $10^{-4}$ level, confirming the method’s accuracy.

The MCS method provides an alternative approach by simulating the real measurement process multiple times, generating input parameters with their actual statistical distributions without assuming normal distribution. In our previous study, this method was validated using both the AutoRFPower software and the Oracle Crystal Ball application. The results showed that the MCS method produced a non-symmetrical normal distribution of uncertainties, reflecting a more realistic representation of the measurement process.

To validate the cloud-based implementation, we conducted uncertainty calculations using the same frequency, power levels, and settings as in the case study presented in Section 4. The results were compared with the manually calculated uncertainties from the original study. This comparison revealed that the cloud-based system consistently yielded results, with the LoP method maintaining its high accuracy and the MCS method accurately representing the non-symmetrical distribution of uncertainties.

The uncertainties determined by both methods were well within acceptable limits, highlighting the effectiveness of the cloud-based system in executing precise and dependable uncertainty calculations. The incorporation of these methods into the microservice architecture guarantees that the system can proficiently handle intricate calibration processes, offering resilient and adaptable solutions for the calibration industry.

### 4.3. Critical Evaluation of Algorithms and Methodologies

In this research, we utilized two different uncertainty calculation methods, the LoP and MCS as outlined by the BIPM [14]. Although LoP is the most commonly used method, it may not be suitable for non-linear models or when input variables deviate significantly from a normal distribution. In such cases, MCS is preferred because it can accurately account for complex interactions and does not require the assumption of linearity or normally distributed inputs, which are often necessary for LoP. Although MCS is more resource intensive, usually requiring at least $10^{5}$ repetitive measurements to simulate real-world conditions, it is particularly valuable in laboratories capable of performing measurements at very high frequencies. Therefore, both methods are employed to leverage their respective strengths in uncertainty analysis.

In the realm of uncertainty calculations, it is important to evaluate the adequacy of various methodologies, from traditional statistical methods like MCS to newer approaches such as predictive machine learning models. While predictive machine learning applications are promising in RF power measurement, they pose particular challenges as well. RF power measurements can be performed with devices from multiple manufacturers, each with unique characteristics that may influence the measurement results. Additionally, the performance of these devices can degrade over time due to wear, which further complicates the task of maintaining the accuracy and reliability of the trained models used in predictive machine learning applications. Environmental factors such as temperature and humidity can also have substantial effects on measurement accuracy. Creating a reliable predictive model for uncertainty calculation requires accounting for these variations through the incorporation of a comprehensive and diverse dataset that encompasses the full range of possible conditions and device behaviors. However, the availability of such data is often limited, as they are considered classified by many institutions, which are reluctant to share them. Obtaining the necessary dataset is challenging, if not impractical, given the number of devices and environmental variables involved. In contrast, the MCS method, which does not rely on historical data or predictive algorithms, provides a more robust approach in this context. MCS can accurately model uncertainty by simulating real-world conditions without the need for extensive training data, making it a more reliable choice for uncertainty calculation in RF power measurements. Nevertheless, machine learning models might offer potential in controlled environments or for specific applications where sufficient data and stable conditions are available.

### 4.4. Results, Implications, Limits, and Future Perspectives

Digitalization efforts in metrology, although still fairly preliminary, are emerging as a critically important endeavor. Metrology as a field is defined by its utmost heterogeneity, consisting of a wide array of complex devices produced by various manufacturers, often lacking interoperability due to proprietary standards, technical challenges and business-related decisions. Additionally, the nature of metrology involves handling sensitive data that demand high computational power and precision during processing, all the while maintaining stringent security measures. Given these unique requirements, the adoption of cloud computing along with microservices presents a particularly apt solution with significant advantages in terms of system flexibility, extendibility, and reduced dependencies through the use of dockerized microservice environments. These characteristics ensure that our system can efficiently integrate diverse components and adapt seamlessly to ongoing technological advancements.

Our cloud-based application incorporates variability handling mechanisms to ensure flexibility and adaptability from a software engineering perspective. Although presently tailored to RF power measurements, the system is inherently extensible and can accommodate a broad spectrum of measurement types, including temperature, pressure, and more. Through systematic variability handling, the application can efficiently manage multiple measurement processes, streamlining configuration and enhancing scalability. Additionally, it offers precise access control, granting users permissions based on their qualifications and eligibility, thus bolstering security.

Our solution emphasizes the benefits of microservice architecture in digital metrology and calibration applications, including scalability and maintainability [38,39]. We aim to drive digital transformation in the metrology and calibration industry by using advanced microservice-based tools and technologies, as traditional monolithic architectures are costly and becoming less effective due to inherent diversity and scalability needs.

Nevertheless, this research may face some limitations that need to be considered. Primarily, the initial cost of cloud technologies may pose a barrier to widespread adoption. However, the long-term benefits such as scalability and operational efficiency are expected to outweigh these concerns. Furthermore, some stakeholders accustomed to handling sensitive data, e.g., in the defense industry, may have reservations about storing their data on the cloud. Even if the cloud-based system has robust security measures, it is still a third-party entity.

Fortunately, our approach is not dependent on any specific Platform as a Service (PaaS) providers. The use of dockerized microservices allows for deployment across various PaaS providers and can be adapted to Infrastructure as Code (IAC), where servers, storage, and networking are managed by developers.This level of flexibility ensures that our system can be tailored to meet the specific security and operational needs of different stakeholders, thereby alleviating some of the concerns associated with cloud adoption.

## 5. Conclusions

This study introduces a microservice-based enhancement to the IoMT architecture, leveraging advanced cloud technologies to support digitalization initiatives in the metrology and calibration industry. The implementation of the AutoRFPower application within this framework showcases the practicality and advantages of integrating automated power measurement processes, LoP- and MCS-based uncertainty calculations, and DCCs into a scalable and maintainable cloud-based system.

Our deployment utilizes Google Cloud to manage containerized assets and microservices, orchestrated by the Google Kubernetes Engine (GKE). The integration of RESTful APIs enables seamless and secure interactions between the client software and cloud services, showcasing the architecture’s efficiency and robustness. The successful deployment and operation of AutoRFPower validate the proposed architecture’s ability to handle the complex demands of metrology and calibration applications.

The microservice-based architecture outlined here offers several key advantages: it enhances system flexibility, reduces dependencies through dockerized environments, and ensures scalability. These attributes are essential for integrating diverse measurement types and adapting to technological advancements, establishing this architecture as a forward-looking solution in the metrology field.

One of the primary challenges in digital metrology lies in the field’s inherent heterogeneity, which is marked by the diversity of devices, standards, technical specifications, and limitations set by various manufacturers. Our proposed solution involves the development of a modular framework that leverages microservices to encapsulate the specific functionalities and standards of different measurement devices. This approach allows for the incorporation of a broad spectrum of measurement types, extending beyond RF power measurement. By encapsulating device-specific operations within independent microservices, we can establish a system capable of accommodating new devices and standards without necessitating substantial changes to the overall architecture.

In the future, our focus will be on expanding the application of the microservice-based architecture to encompass other calibration processes, such as the scope of accreditation. By adhering to the proposed architecture, these processes can similarly benefit from the scalability, flexibility, and maintainability offered by microservices. Furthermore, further research will explore ways to maximize the advantages of microservice-based solutions, potentially incorporating additional functionalities and optimizing performance across various metrological applications.

We are also exploring the incorporation of advanced analytics and machine learning models to bring predictive capabilities to the system. This may involve developing algorithms that anticipate equipment maintenance needs and intervals, optimize measurement procedures, or detect anomalies in real-time. Additionally, we will investigate the potential for interoperability with other emerging digital metrology systems, aiming to create a more connected and cohesive ecosystem. This may include collaborating with industry partners to establish standardized interfaces and protocols.

Overall, this study highlights the transformative potential of microservice-based cloud architectures in the metrology and calibration industry. By tackling current challenges and paving the way for future advancements, our work establishes a foundation for ongoing innovation and improvement in digital metrology practices.

## Data Availability

Data are contained within the article.

## Abbreviations

The following abbreviations are used in this manuscript:

BIPM	International Bureau of Weights and Measures
BPMN	Business Process Model and Notation
CI/CD	Continuous Integration/Continuous Delivery
CLs	Calibration laboratories
CSAPI	Cloud Services API
DCC	Digital Calibration Certificate
DCCs	Digital Calibration Certificates
GKE	Google Kubernetes Engine
GPIB	General Purpose Interface Bus
GUM	Guide to the Expression of Uncertainty in Measurement
IaC	Infrastructure as Code
IEEE	Institute of Electrical and Electronics Engineers
IIoT	Industrial Internet of Things
IoMT	Internet of Measurement Things
LoP	Law of Propagation
MAC	Media Access Control
MCS	Monte Carlo Simulation
MII	Metrology Information Infrastructure
NI	National Instruments
NUM	NIST Uncertainty Machine
PaaS	Platform as a Service
PDFs	Probability Density Functions
PTB	Physikalisch-Technische Bundesanstalt
QR	Quick Response
ScoA	Scope of Accreditation
SoA	Service-oriented Architecture
TVM	Textual Variability Model
UI	User Interface
UML	Unified Modeling Language
UUID	Universally Unique Identifier
